# Design and development of patient health tracking, monitoring and big data storage using Internet of Things and real time cloud computing

**DOI:** 10.1371/journal.pone.0298582

**Published:** 2024-03-11

**Authors:** Imran Shafi, Sadia Din, Siddique Farooq, Isabel de la Torre Díez, Jose Breñosa, Julio César Martínez Espinosa, Imran Ashraf

**Affiliations:** 1 College of Electrical and Mechanical Engineering, National University of Sciences and Technology, Islamabad, Pakistan; 2 Texas A&M University at Qatar, Ar-Rayyan, Qatar; 3 National Centre for Robotics and Automation (NCRA), National University of Sciences and Technology (NUST), Islamabad, Pakistan; 4 Department of Signal Theory and Communications and Telematic Engineering, University of Valladolid, Valladolid, Spain; 5 Universidad Europea del Atlántico, Santander, Spain; 6 Universidad Internacional Iberoamericana Campeche, México; 7 Universidad Internacional Iberoamericana, Arecibo, Puerto Rico, United States of America; 8 Universidade Internacional do Cuanza. Cuito, Bié, Angola; 9 Fundación Universitaria Internacional de Colombia, Bogotá, Colombia; 10 Department of Information and Communication Engineering, Yeungnam University, Gyeongsan, South Korea; KUET: Khulna University of Engineering and Technology, BANGLADESH

## Abstract

With the outbreak of the COVID-19 pandemic, social isolation and quarantine have become commonplace across the world. IoT health monitoring solutions eliminate the need for regular doctor visits and interactions among patients and medical personnel. Many patients in wards or intensive care units require continuous monitoring of their health. Continuous patient monitoring is a hectic practice in hospitals with limited staff; in a pandemic situation like COVID-19, it becomes much more difficult practice when hospitals are working at full capacity and there is still a risk of medical workers being infected. In this study, we propose an Internet of Things (IoT)-based patient health monitoring system that collects real-time data on important health indicators such as pulse rate, blood oxygen saturation, and body temperature but can be expanded to include more parameters. Our system is comprised of a hardware component that collects and transmits data from sensors to a cloud-based storage system, where it can be accessed and analyzed by healthcare specialists. The ESP-32 microcontroller interfaces with the multiple sensors and wirelessly transmits the collected data to the cloud storage system. A pulse oximeter is utilized in our system to measure blood oxygen saturation and body temperature, as well as a heart rate monitor to measure pulse rate. A web-based interface is also implemented, allowing healthcare practitioners to access and visualize the collected data in real-time, making remote patient monitoring easier. Overall, our IoT-based patient health monitoring system represents a significant advancement in remote patient monitoring, allowing healthcare practitioners to access real-time data on important health metrics and detect potential health issues before they escalate.

## Introduction

Internet of Things (IoT) is defined as things having identities and virtual personalities operating in smart spaces using intelligent interfaces to connect and communicate within social, environmental, and user contexts. It can be considered the future of the Internet, where every object is connected to other objects. The network assigns each thing a distinct identification. This enables devices to be accessed remotely via the network at any time and from anywhere. Smart, pervasive, and always-connected environments are created by IoT-equipped things communicating with one another, accessing information through the Internet, and interacting with users.

Physicians and healthcare workers have access to large databases of medical information as well as a variety of tools, such as artificial intelligence (AI) and machine learning, to enhance patient treatment and results. Patient monitoring, on the other hand, is one component of patient care that has yet to benefit from technology advancements. Patient monitoring using the traditional approach is a difficult task that necessitates extra manpower on the other hand Health professionals are more likely to overlook significant changes in patient vitals while such as blood pressure, pulse rate, and oxygen saturation. In Pakistan, the nurse-to-patient ratio is 1:50 [1], compared to a standard of 1:5. As per World Health Organization, Pakistan has 5 nurses and midwives for every 10,000 people, compared to 10 medical professionals whereas the internationally recommended doctor-to-nurse ratio is 4:1. Hospitals require a smart system that can track patients’ physiological changes in order to manage huge populations of patients with limited healthcare staff. The increased usage of mobile technology and smart devices has had a significant impact around the world. Health professionals are rapidly recognizing the advantages of these technologies, resulting in considerable improvements in clinical care [2]. Early identification, preventive, and protracted management of medical disorders are becoming more common in healthcare, which is evolving from a reactionary to a proactive approach [3]. The monitoring system enables a user to closely monitor and give feedback on fluctuations in physiological parameters, assisting in the retention of good health.

Intrahospital movement of patients is becoming increasingly common in order to perform specialized diagnostics or therapies [4]. Continuous health monitoring, such as ECG, oxygen level/saturation, pulse rate, and blood pressure is important to the effectiveness of any critical care transport [5]. Being aware of the vital changes that occur when patients are transferred aids in the formulation of a preventative and early diagnosis and treatment strategy that assures a safe and comfortable journey. Health-sensing components have shrunk in size and weight, allowing patients to monitor their health around the clock. It functions as an information retrieval system, transferring data from the physical to the digital realms. A virtual patient in the digital world is an IoT-enabled health monitoring gadget connected to a patient [6]. The physiological circumstances of the virtual patient are identical to those of the real one. Because of a spate of new healthcare technology start-ups, IoT is quickly altering the healthcare sector. Alternatives to normal patient management are available through the health monitoring system. Additionally, this technology reduces healthcare expenditures and supports the hospital in enhancing the treatment process while also providing a remote health-monitoring system. Patients’ health care and doctors’ professions can both benefit from technological advances. The health monitoring system is an excellent example of how current technology is implemented in hospitals. A Health Monitoring System is a cutting-edge technology that can help nurses and doctors, as well as patients at home, with the traditional patient and health management.

The proposed IoT-based health monitoring system represents a significant contribution to the healthcare field, especially during the COVID-19 pandemic. It offers a scalable solution for continuous patient monitoring, eliminating the need for regular doctor visits and interactions between patients and medical personnel, and ultimately reducing the risk of infection transmission. The system’s ability to lessen the workload of healthcare practitioners in hospitals with limited staff, especially during the pandemic, highlights its potential to have a positive impact on patient care and outcomes. Thus, the proposed system has the potential to improve the quality of patient care and outcomes while mitigating the risks associated with the pandemic.

The rest of the paper is organized into four sections. “Literature Review” section discusses several works related to the current study. The proposed approach is presented in the “Proposed Methodology” section. Results are discussed in the “Results and Discussions” section while the “Conclusion” section presents the conclusion.

## Literature review

With the spread of the COVID-19 pandemic, social isolation and quarantine have become prevalent worldwide. IoT health monitoring solutions eliminate the need for regular medical appointments and interactions among patients and medical personnel. Even though, many people require medical experts to examine and observe their health on a frequent basis. IoT-based smart health monitoring system [7] is proposed to address these issues, proficient in measuring patient’s temperature, heart rate, and oxygen saturation. If any abnormal values do not meet the standard values an alert is generated and sent to the concerned medical staff. Tati Erlina et al. [8] developed a system for monitoring an unconscious patient’s physiological condition by assessing pulse rate, respiration rate, and eyelid status This entire system is intended to communicate data to a smartphone app, which is designed to display various readings. The strategy, however, failed to ensure system security, placing important data at risk.

The Internet of Things (IoT) has the potential to improve healthcare. Doctors in an emergency might engage with health-related data. The doctor may know the patient’s state even if he or she is not nearby or in the hospital so that in emergency circumstances, the doctor’s advice can be offered. Long et al. [9] addressed the essential and required elements of healthcare systems and presented a healthcare and IoT architecture that can record an ECG, blood oxygen saturation, respiration, and body temperature When a patient’s condition deteriorates, it generates an alert. [10]. To examine data, Node MCU is used as a microcontroller, and notifications are delivered to physicians and concerned individuals via email and Twitter. It also stores and keeps track of previous patient medical records about the patient’s condition. The patient’s current status is relayed to medical specialists via an internet portal, and the proper medication may be given to cure the sufferer.

In comparison to wired applications, wireless communication is preferred since it requires less maintenance effort/cost and is simpler. The research of Lei Ru [11] focuses on a variety of wearable health monitoring modules that people use to measure their and other physiological data. A health monitoring device is present, which sends the observed physiological parameters to several computers via a wireless communication unit [12]. To improve healthcare service quality, an IoT-based monitoring system in the Intensive Care Unit (ICU) is proposed [13]. This paper’s concept focuses on the effective monitoring of multiple activities (including anomalies) with transitory dependencies, accompanied by time-sensitive warnings. The findings reveal that IoT-enabled ICUs are far more effective than manual and traditional Tele-ICU monitoring at monitoring important events. Furthermore, the approach for generating alerts gives additional information and enhances the system’s benefits. Medical information such as Sp02, pulse rate, and blood pressure is critical in determining an individual’s or a patient’s condition in a healthcare context [14]. They provide very significant information regarding their present state of health, and thus facilitating the process of obtaining such data with sensors has significant benefits [15].

There is a home monitoring and decision support system to help doctors diagnose Parkinson’s disease, remote patient monitoring, pharmacological treatment, prescriptions, therapy, and progression. This system collects data about tremors and assists doctors in diagnosing and treating them [16]. Another system, known as the Remote Patient Monitoring System (RPMS), attempts to administer hospital resources by screening patients at home [17]. RPMS is an IoT-based health monitoring system that facilitates the acquisition and transfer of patient information to remote databases automatically. This data is accessible via a web platform with a user-friendly GUI.

Bhoomika proposed a healthcare system that monitors heart rate and body temperature utilizing a PIC18F46K22 microcontroller, DS1820B temperature sensor, and MCP6004 pulse oximeter and heart rate sensors [18]. The microcontroller accesses the data and transmits it via the ESP8266 Wi-Fi protocol. The data is displayed on the LCD so that the patient is informed of his current health status. During an emergency, a message is sent to the doctor’s mobile phone through the GSM modem, and the buzzer sounds to warn the caregiver. The doctors may access the data by entering into the website using a unique IP address and reloading the page, resulting in continuous data receiving. A similar system that collects the patient’s vital signs, such as pulse rate, blood pressure, pulse oximetry, and temperature, is proposed [19] that collects vital signs from the patient, such as pulse rate, blood pressure, pulse oximetry, and temperature There was no real-time ECG signal in this investigation. An Arduino Yun is used as the microcontroller board to record and analyze the data. The data is subsequently sent to the cloud via WLAN using an Application Programming Interface (API). Doctors can access the webserver to review the patient’s medical information and make remarks to the patient. The data is momentarily saved on the SD card if the device loses its WLAN connection. And once the server connection is restored, the system automatically updates the data with the server. This system lacked a real-time ECG signal.

Proof of concept of a remote patient monitoring system is presented by Mustapha et al. [20] that uses Edge Cloud and Web Real-Time Communication (WebRTC). WebRTC is employed as a significant architectural element in intelligent health environments to facilitate interactive connections with remote users. Edge Cloud is used to reduce network bandwidth consumption, particularly for video traffic for remote monitoring and management of medical equipment in real time. There are two modes to the system: push and pull. If suspicious health data is identified in a push-mode monitoring system, the system automatically sends a notification to the user. Monitoring systems in pull mode allow the user to examine previously acquired data. Literature gaps in IoT-enabled patient health monitoring systems include usability and the ability to provide real-time response. Many systems currently available are complicated to use and require extensive training, making them inaccessible to patients and healthcare providers with limited technical expertise. Additionally, many systems rely on passive monitoring, which may not detect emergencies or critical events in real-time. Therefore, there is a need to develop user-friendly interfaces that are intuitive and easy to use and active monitoring systems that can alert healthcare providers or emergency responders in real-time when critical events occur. In terms of medical diagnostics, it is evident that expensive and advanced medical tools give excellent service to users. The reality that people in underdeveloped nations usually lack access to such expensive medical devices for adequate treatment owing to their countries’ social-economic framework is also apparent. As a result, the design and development of low equipment based on current technology must be prioritized in order to ensure that every patient has access to high-quality medical care. By considering the problem stated above and literature gaps identified, there is a pressing need for a system that can monitor patients’ health parameters in real time, thereby eliminating the need for constant manual monitoring in a hospital setting. To address this need, a portable and comprehensive patient monitoring system based on IoT technology has been proposed. This system has the capability to not only monitor the patient’s vital signs in real time but also store the data for record-keeping purposes. Furthermore, if any abnormalities are detected by the system, it can notify either the patient or their doctor accordingly. The proposed system is designed to be user-friendly, such that no extensive training is required to operate it. This feature has been incorporated to ensure that the system can be easily used by patients or caregivers without any technical expertise.

## Proposed methodology

**Fig 1:**
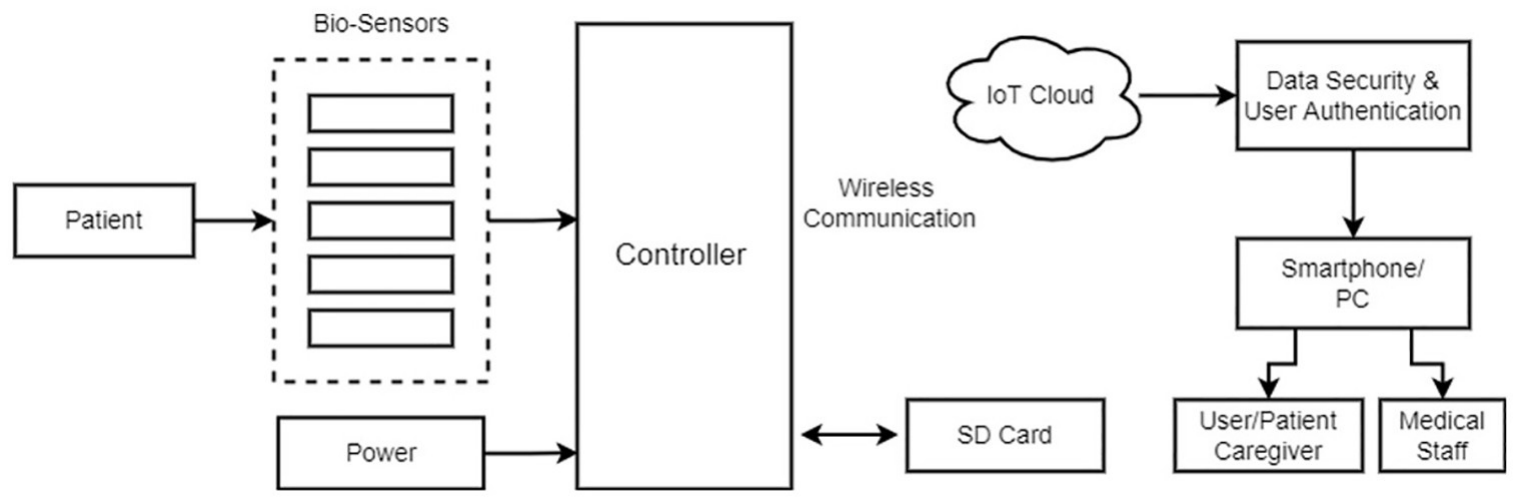
Block diagram of the proposed approach.

The working for projects is divided into two parts. The first part surrounds the interfacing of sensors being used in the system. Testing and implementing these hardware components with a microcontroller acting as the base processor to process all inputs. The second part then deals with storing, displaying, and notifying the data coming from the sensors. Cloud-based services offer a significant benefit in this IoT process. A basic block diagram is shown in Fig 1.

The physiological parameters of the patient are monitored using biosensors that are integrated with the controller, as illustrated in the block diagram. The data is subsequently transmitted to the IoT cloud, where it is stored. Data may be monitored from anywhere in the globe using password-protected credentials that make it safe and secure. If the cloud connection is lost, data is temporarily kept on the SD card and uploaded to the cloud when the connection is restored.

### Ethics statement

The study was reviewed and approved by the National University of Sciences and Technology (NUST) Institutional Review Board (IRB) in Islamabad, Pakistan. This case study included patients with hypertension, diabetes, and other related medical conditions. Each patient provided written informed consent to participate in the study. Patient confidentiality has been ensured, and their data will remain anonymous.

### Experimental setup

**Fig 2:**
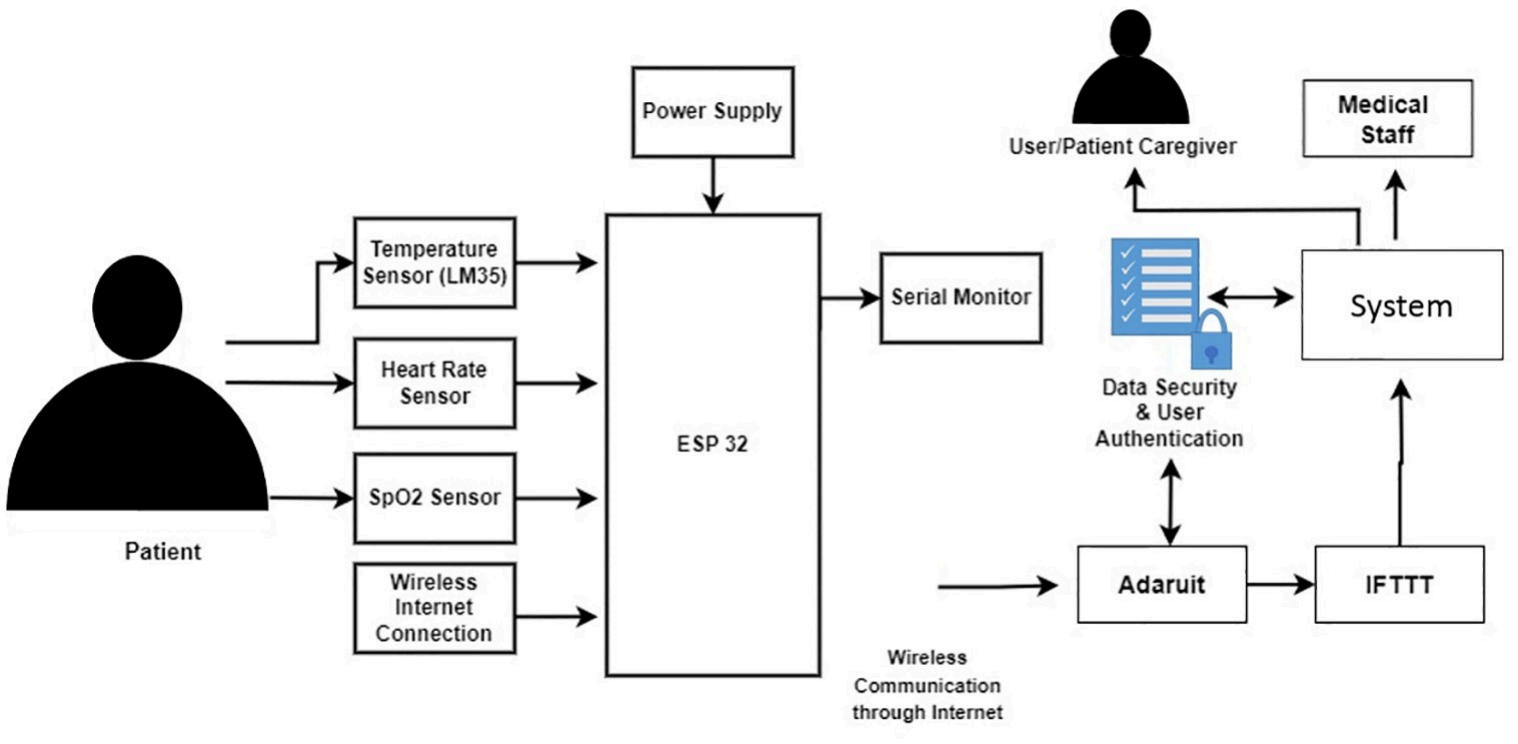
Prototype design.

A portable prototype experimental setup is designed to address critical parameters. The MQTT (message queue telemetry transport) protocol is used to communicate data between the controller and the cloud server. Dashboard and mobile application then receive data from the cloud server. Adafruit IO is used for storing data in a cloud database from where it can be easily accessible and downloadable with a single click, and also for displaying real-time data on a dashboard. Furthermore, IFTTT is connected with Adafruit for sending notifications/message alerts to the caregiver and medical concerned staff. The basic Prototype design is shown in Fig 2.

An oximeter is used to measure heart rate and oxygen saturation and the model here is SO2 MAX30102. The LM35 temperature sensor is used to monitor body temperature. As LM35 is an analog sensor so GPIO 36 of ESP32 i.e ADC1 CH0 is connected with the output pin of LM35. An oximeter is integrated with ESP 32 microcontroller by allotting D22 and D21 pins for the SDA and SCL of the oximeter for the l2C protocol. Ground and Vcc are given to respective pins by the microcontroller.

**Fig 3:**
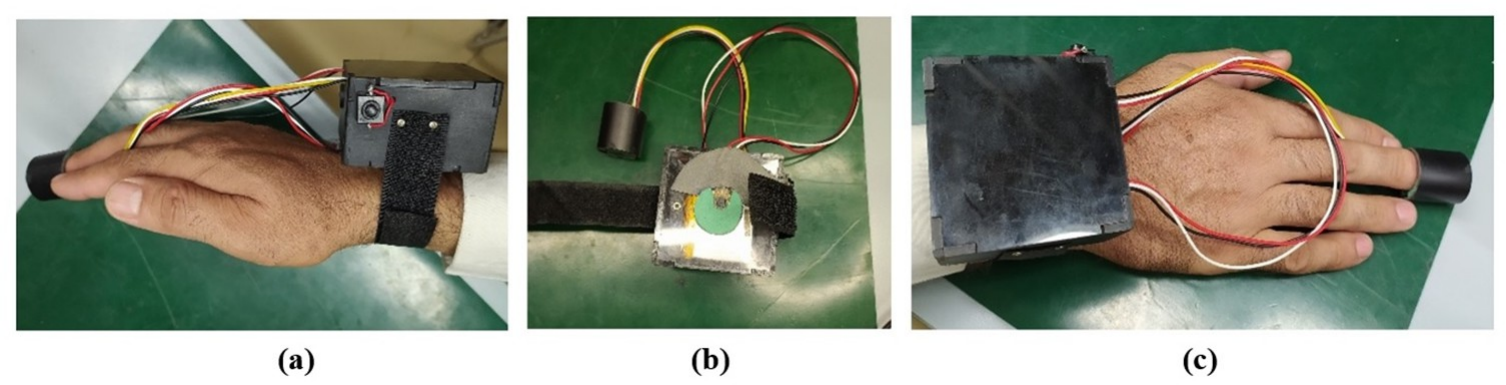
(a) Wearable device for patient health monitoring (b) LM35 on the bottom side (c) Finger strap containing Oximeter.

The system was integrated into a wearable device as shown in Fig 3a. The device shown contains all the circuitry inside it for patient health monitoring. The wearable device has a strap for wearing on the wrist and therefore, it is portable. It is powered by 5 Volts from a rechargeable battery integrated into the device. There is a charging slot at the side for recharging the battery and a power button to turn off the device for battery saving whenever the device is not being used. The LM35 temperature sensor is mounted on the bottom of the device, and insulation is also integrated between the device and LM35 to avoid interference from the self-heating of the device as shown in Fig 2 and the Oximeter as shown in Fig 3c is mounted on the inside the finger strap. The Oximeter is enclosed so that no ambient light disturbs the measurements because of the fact that it works on the principle of spectrophotometry, which is sensitive to ambient light. By putting a finger on the oximeter, it will measure the heart rate and oxygen saturation level in the blood, and the body temperature is given by LM35. Data can be read through a serial monitor and also on the ADAFRUIT IO dashboard and smartphone application. Red and IR LEDs in an oximeter measure the heart rate. The data received now will be shown on some networks where real-time data can be displayed and stored. Adafruit is used for displaying the data as it is most widely being used and is more effective and easier to use.

The feed from the oximeter in the form of temperature and heart rate, oxygen saturation will be uploaded to Adafruit IO through MQTT. This data will be displayed on Adafruit IO and smartphone application. Now using the dashboard, we can see our data in graphical or any other form. Heart rate and temperature values will be shown in real-time.

As the data is being recorded and displayed, Adafruit is further connected to IFTTT. This means that if a certain event occurs, then a specific action should be taken. The data is now being constantly monitored. The normal heart rate of an adult typically ranges between 60-100 beats per minute, with some variations that can lie in the range of 40-120. If the heart rate of the person goes below or above the defined range, the system will trigger a notification on the mobile device, indicating that the user requires assistance.

## Results and discussions

**Fig 4:**
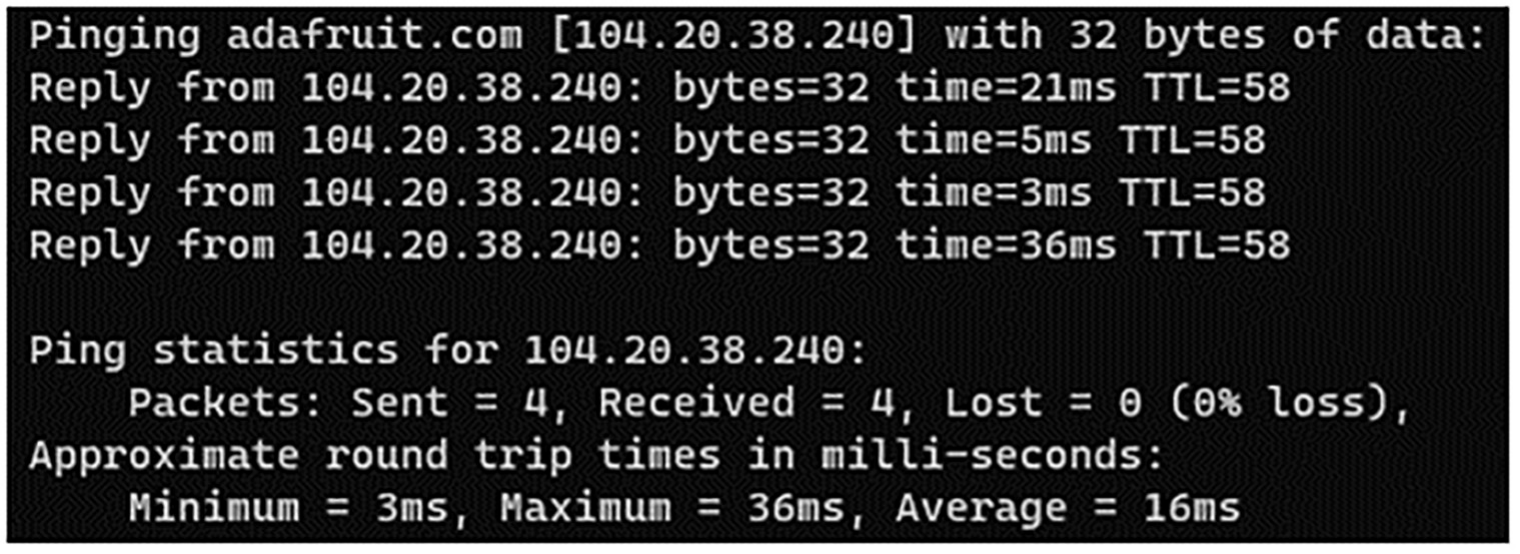
Latency of IoT server.

After initializing controller and sensors are mounted on the Patient’s Wrist and finger. To get a visual representation of data it takes a few seconds to get the stable value from the sensors to be displayed after the controller is initialized as shown in Fig 4. Average power consumption of various components is given in Table 1.

**Table 1:** Average power consumption.

Component	Avg Current (mA)
ESP-32	180mA
Pulse Oximeter	20mA
Total Current	200mA

The latency of IoT platform can vary depending on various factors such as the location of the server, network conditions, and the volume of traffic on the platform. However the average latency of proposed system that we measured is 16ms as shown in the Fig 4 Response time for IoT platform is 1 sec, as the limit of Adafruit is 60 data points per minute.

The pulse oximeter contains the heart rate, body temperature and, oxygen saturation measurement. The sensor has been chosen carefully due to its low power consumption.

**Fig 5:**
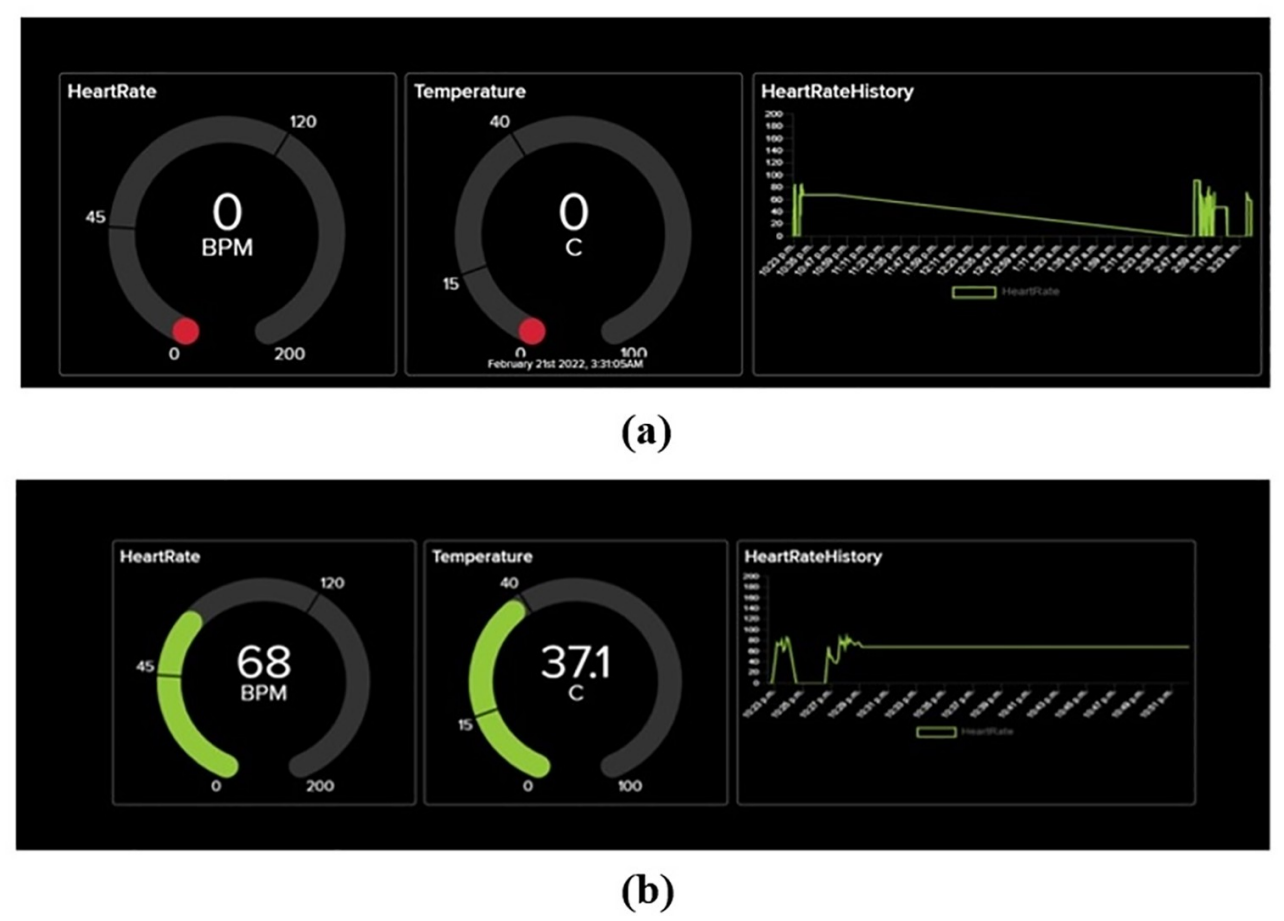
Real-time patient vitals.

Both temperature data and heart rate can be seen in real-time Fig 5b.

IFTTT algorithm implementation and receiving the notification are carried out. Creating a simple AI algorithm on IFTTT and setting heart rate limits to receive critical situation alerts. It keeps check on all patient vitals and whenever they fall below or shoot above optimal range an alert is sent to concerned personnel (i.e. medical staff & Patient caregivers).

**Fig 6:**
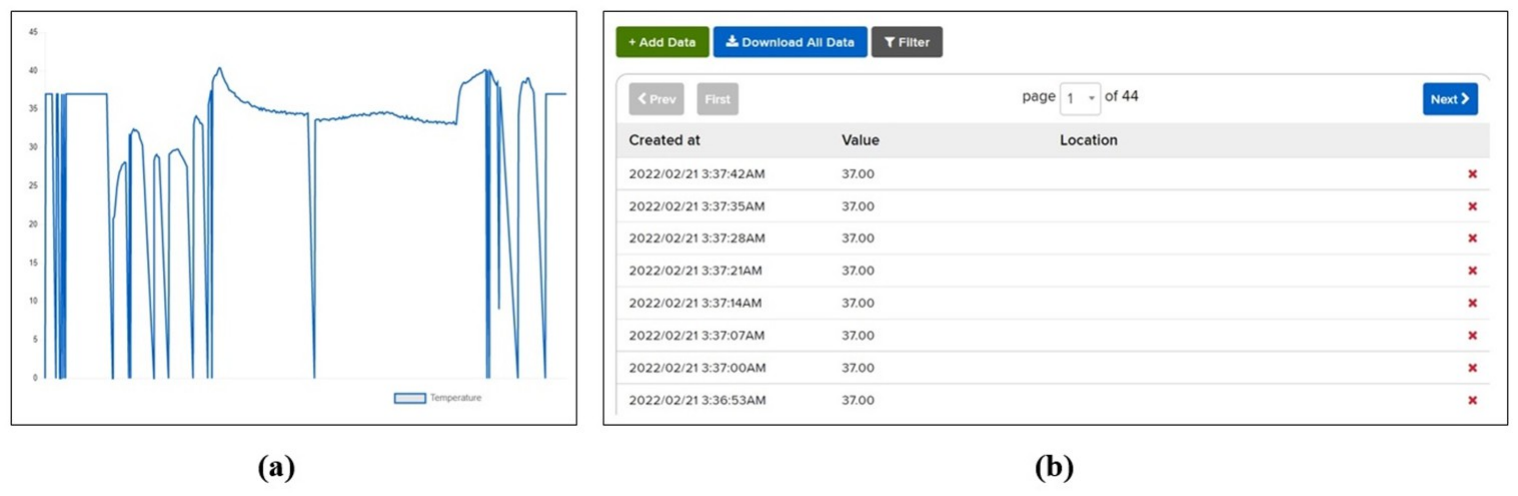
Record of patient body temperature over time.

**Fig 7:**
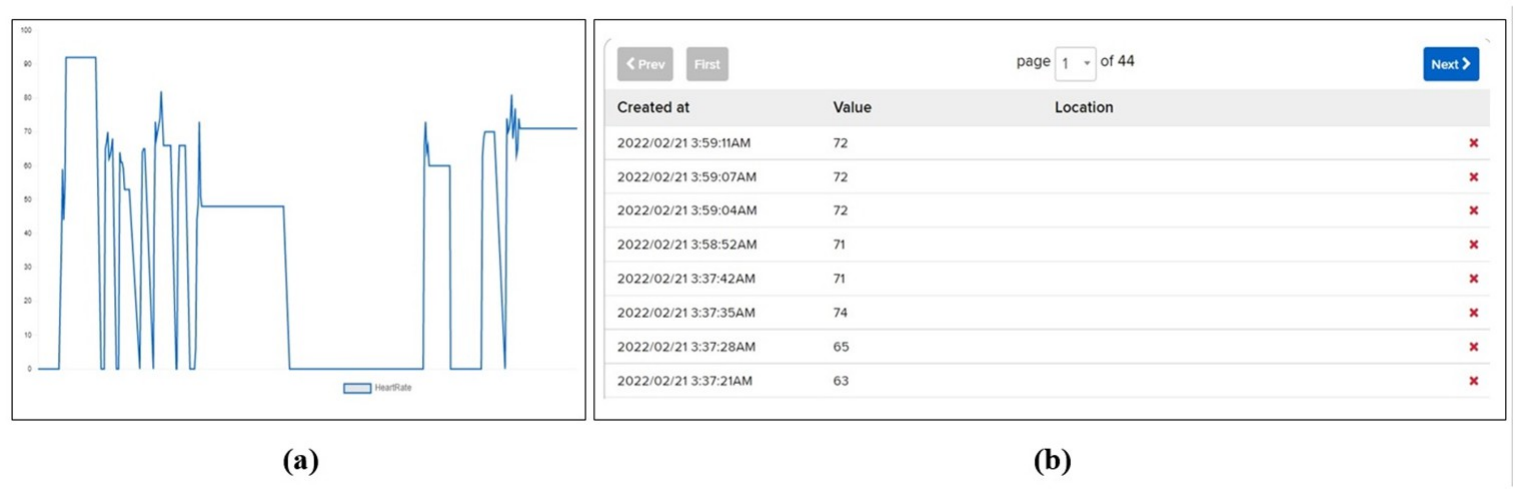
Record of patient heart rate over time.

Other than displaying patient vitals, this system is capable of storing the history of patient vitals and mapping it across time to show changes in vital signs graphically. Fig 6 shows the record of the patient body temperature over time. It proves helpful in diagnosis as it is impossible to continuously monitor patients’ conditions. Similarly, other vitals from a patient can be recorded like the heart rate over a period of time which can be recorded and analyzed when needed. For example, Fig 7 shows the record of a patient heart rate over a specific period of time as recorded by the proposed system. Moreover, history can be downloaded for medical records or used as a case study with a single click.

**Fig 8:**
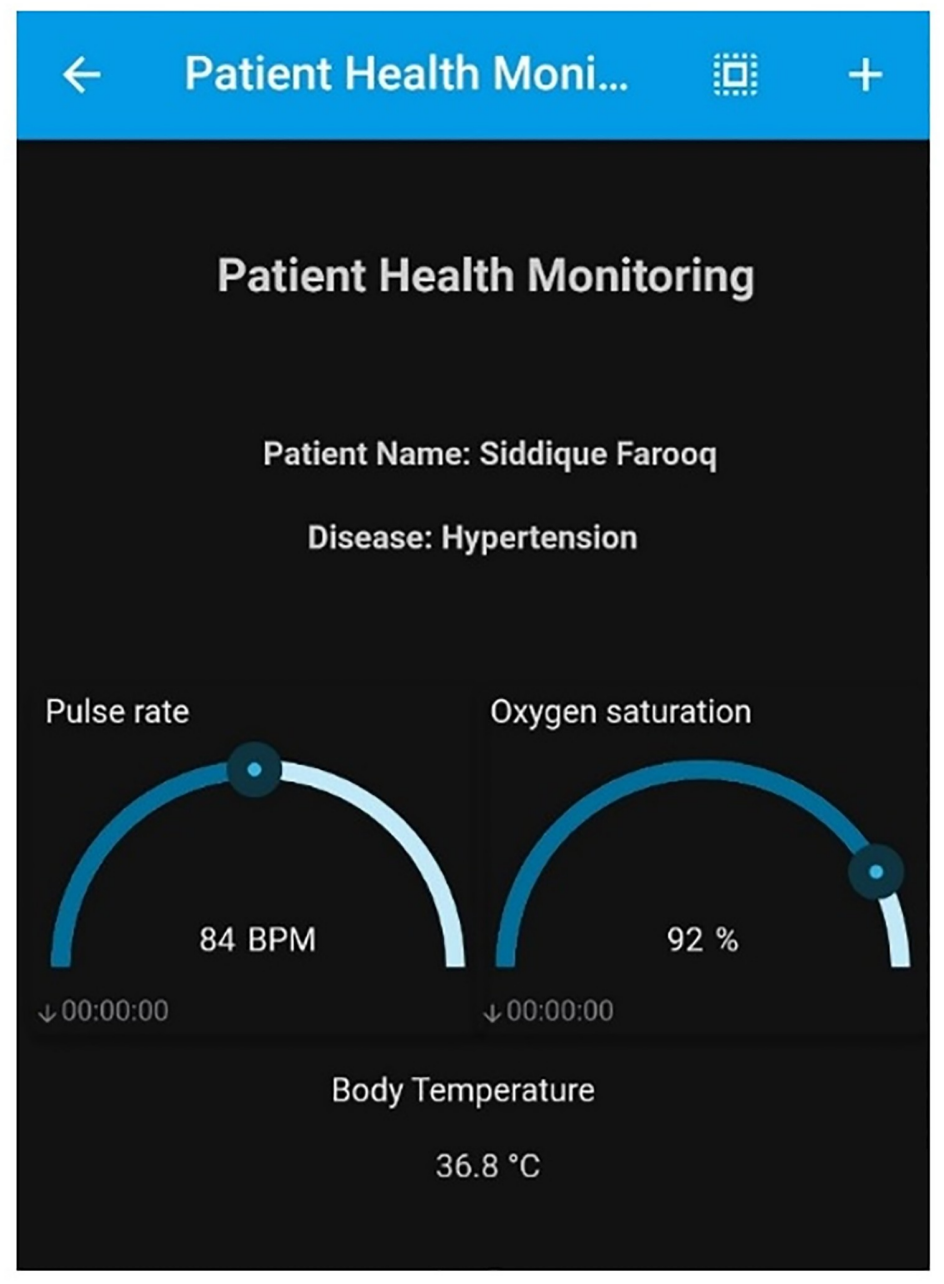
Snapshot of application showing pulse rate, oxygen saturation & body temperature.

Depending on the hospital’s policy and the patient’s privacy concerns, data feeds might be kept private or made public. Such settings can be made using the setting options provided with the proposed system. Administrators have access to add concerned personnel to the feed as an admin or user. Admins have full access to the data feed to read, write and share the feed whereas the users can only view it. A snapshot of the smartphone application showing patient vitals is shown in Fig 8. The developed app provides a real-time view of the pulse rate, temperature, oxygen saturation level, etc. of a patient.

To evaluate the efficiency of the proposed system, it is tested on several subjects of different age groups and the results are recorded as observed values. Reference values for heart rate, oxygen saturation level in the blood, and body temperature were obtained using manual equipment and users already in use in the hospital and are written as actual values. Actual and observed data along with percentage error for body temperature, oxygen saturation level in the blood, and pulse rate are shown here. Table 2 shows the actual and observed temperature using the proposed approach. Similarly, the error in the observed and actual observation of oxygen saturation is displayed in Table 3.

**Table 2:** Actual temperature measured with an analog thermometer and proposed system (Observed).

Subjects	Actual temperature (°C)	Observed temperature (°C)	Error
1	36.3	36.2	0.1
2	36.5	36.7	0.2
3	36.2	36.1	0.1
4	36.7	36.8	0.1
5	36.2	36.4	0.2

**Table 3:** Oxygen saturation measured by a commercially available device (Actual) and proposed system (Observed).

Subjects	Actual SaO_2_ (%)	Observed SaO_2_ (%)	Error (%)
1	94	92	2.12
2	97	96	1.03
3	96	98	2.04
4	98	97	1.02
5	92	95	3.00

Table 4 shows the heart rate, both actual and observed, along with the percentage error from the proposed approach.

**Table 4:** Actual pulse rate measured by analog machine and proposed system (Observed).

Subjects	Actual (BPM)	Observed (BPM)	Error (%)
1	75	76	1.31
2	81	83	2.40
3	85	84	1.17
4	96	98	2.04
5	82	83	1.20

**Fig 9:**
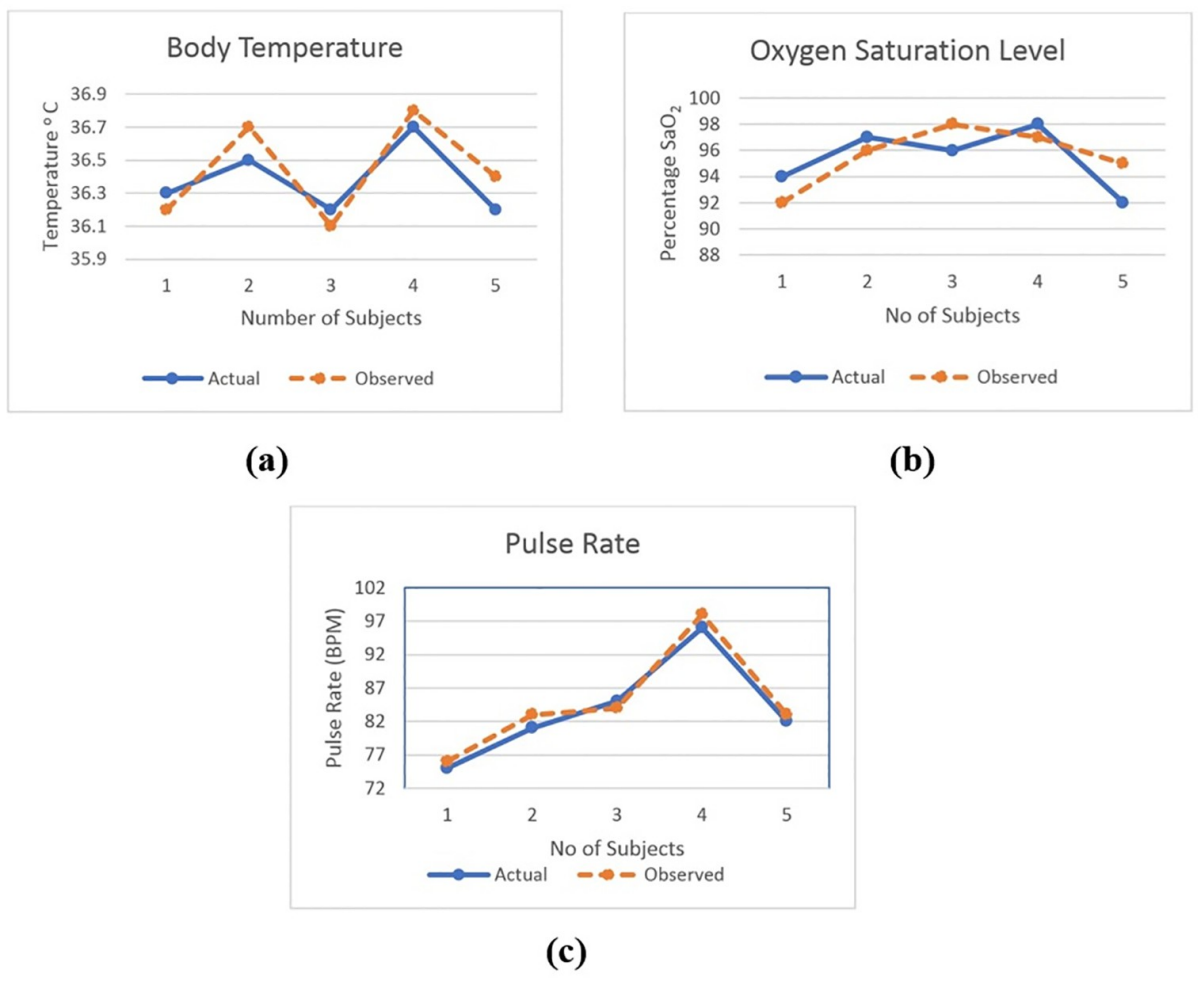
Results of actual and observed values for (a) Body temperature, (b) Oxygen saturation level, and (c) Pulse rate.

To assess the difference between the data obtained by the designed system and the actual data Fig 9 is shown here. The real and observed results differ slightly. The data for body temperature is shown in Fig 9a. The sensor’s inaccurate placement and the effects of the environment are discovered to be the causes of the variation in body temperature. Fig 9b represents the actual and observed values of oxygen saturation level in blood. Actual values are obtained using a manual device that is already in use in hospitals. The deviation of actual and observed pulse rates of 5 different individuals are shown in Fig 9c. The deviation in pulse rate and oxygen saturation level occurred as a result of motion artifacts generated by subject movement during treatment. Sometimes when the sensor is misplaced, it gives erroneous data. Furthermore, it is also caused by the light scattering from other sources.

**Fig 10:**
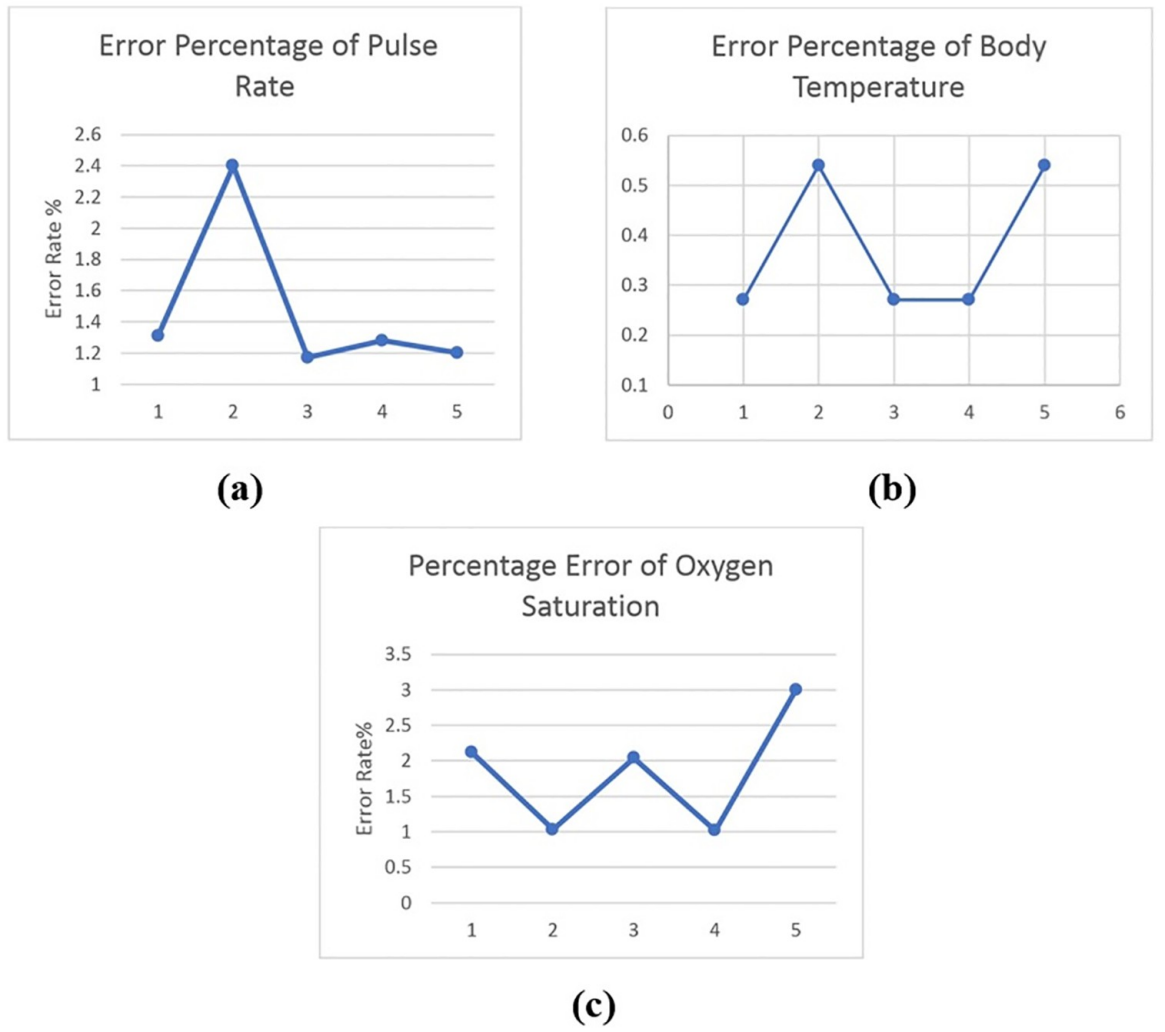
Error percentage of the proposed system for, (a) Body temperature, (b) Oxygen saturation level, and (c) Pulse rate.

Fig 10a–10c show the system’s error rate for measuring body temperature, pulse rate, and oxygen saturation level, respectively. The highest error rates are 0.54%, 2.04%, and 2.4%, respectively, and the lowest are 0.27%, 1.02%, and 1.17%. All of the errors are less than 5%, which is considered acceptable.

Table 5 shows the performance comparison of our proposed system with those in literature.

**Table 5:** Performance comparison.

Reference	Controller Used	Health Parameters	Portability	Error (%)
[10]	ESP32	Heart Rate, Body temperature, Room Humidity,	Yes	Less than 5%
[3]	PIC micro-controller	Electrocardiogram, Body temperature	No	Not provided
[7]	Arduino Uno, Node MCU	Body temperature, pulse rate and SPO2	No	Not provided
[21]	Arduino Uno, Bluetooth, WiFi modules	Blood Presure, Heart rate	No	Not Provided
Proposed Solution	ESP32	Body temperature, Pulse rate, SpO2	Yes	Less than 3%

## Conclusion

This study presents an intelligent health monitoring system that makes it simple for doctors to examine patients’ vitals on a smartphone or a computer at their office without personally visiting them. Aside from the doctor, the patient is constantly monitored by an intelligent system, and if vitals are above or below the normal range, an alert is generated and broadcast to the concerned medical staff and the patient’s caregiver. Data is continually uploaded to the cloud, and if the connection to the cloud is interrupted for any reason, data is temporarily kept on an SD card and uploaded to the cloud the instant the connection is restored. Real-time data is displayed on the feed and is available to medical staff and can be shared with the Patient’s caretaker as well. No one can view feed other than concerned persons or persons to whom feed is shared. This makes it a secure system. As seen in the results, the data is correctly displayed while also being saved on the cloud as a history of the patient. Doctors can make a comparison between a patient’s prior values and the current ones based on their data. History can be downloaded for medical records or can be used as a case study. This approach can help patients save time and money by reducing their commute time and costs, particularly for individuals who live in suburban or rural locations. In the event of infectious diseases, such as coronavirus (COVID-19), the system is quite beneficial as it allows contactless patient monitoring, doctors can examine patients’ vitals without personally visiting them inwards hence providing safety to medical staff and allowing them to monitor multiple patients at the same time that was the hectic task before.
